# Co-Expression of Niemann-Pick Type C1-Like1 (NPC1L1) with ACE2 Receptor Synergistically Enhances SARS-CoV-2 Entry and Fusion

**DOI:** 10.3390/biomedicines12040821

**Published:** 2024-04-08

**Authors:** James Elste, Nicole Cast, Shalini Udawatte, Kabita Adhikari, Shannon Harger Payen, Subhash C. Verma, Deepak Shukla, Michelle Swanson-Mungerson, Vaibhav Tiwari

**Affiliations:** 1 Department of Microbiology and Immunology, Midwestern University, Downers Grove, IL 60515, USA; jelste@midwestern.edu (J.E.); nicole.cast@midwestern.edu (N.C.); mswans@midwestern.edu (M.S.-M.); 2 School of Chemistry & Biochemistry, Georgia Institute of Technology, North Ave NW, Atlanta, GA 30332, USA; shalinisam101@gmail.com; 3 Department of Microbiology & Immunology, University of Reno, Reno, NV 89557, USA; kadhikari@unr.edu (K.A.); shannonharger@med.unr.edu (S.H.P.); scverma@med.unr.edu (S.C.V.); 4 Department of Microbiology and Immunology, University of Illinois, Chicago, IL 60612, USA; dshukla@uic.edu

**Keywords:** viral entry, virus cell fusion, ACE2, NPC1L1

## Abstract

The entry of severe acute respiratory syndrome coronavirus 2 (SARS-CoV-2) into human embryonic kidney (HEK293T) cells has been shown to be a cholesterol-rich, lipid raft-dependent process. In this study, we investigated if the presence of a cholesterol uptake receptor Niemann-pick type c1-like1 (NPC1L1) impacts SARS-CoV-2 cell entry. Initially, we utilized reporter-based pseudovirus cell entry assays and a spike (S) glycoprotein-mediated cell-to-cell fusion assay. Using Chinese hamster ovary (CHO-K1) cells, which lack endogenous receptors for SARS-CoV-2 entry, our data showed that the co-expression of NPC1L1 together with the ACE2 receptor synergistically increased SARS-CoV-2 pseudovirus entry even more than the cells expressing ACE-2 receptor alone. Similar results were also found with the HEK293T cells endogenously expressing the ACE2 receptor. Co-cultures of effector cells expressing S glycoprotein together with target cells co-expressing ACE-2 receptor with NPC1L1 significantly promoted quantitative cell-to-cell fusion, including syncytia formation. Finally, we substantiated that an elevated expression of NPC1L1 enhanced entry, whereas the depletion of NPC1L1 resulted in a diminished SARS-CoV-2 entry in HEK293T-ACE2 cells using authentic SARS-CoV-2 virus in contrast to their respective control cells. Collectively, these findings underscore the pivotal role of NPC1L1 in facilitating the cellular entry of SARS-CoV-2. Importance: Niemann-Pick type C1-like1 (NPC1L1) is an endosomal membrane protein that regulates intracellular cholesterol trafficking. This protein has been demonstrated to play a crucial role in the life cycle of several clinically important viruses. Although SARS-CoV-2 exploits cholesterol-rich lipid rafts as part of its viral entry process, the role of NPC1L1 in SARS-CoV-2 entry remains unclear. Our research represents the first-ever demonstration of NPC1L1’s involvement in facilitating SARS-CoV-2 entry. The observed role of NPC1L1 in human kidney cells is not only highly intriguing but also quite relevant. This relevance stems from the fact that NPC1L1 exhibits high expression levels in several organs, including the kidneys, and the fact that kidney damages are reported during severe cases of SARS-CoV-2. These findings may help us understand the new functions and mechanisms of NPC1L1 and could contribute to the identification of new antiviral targets.

## 1. Introduction

Severe acute respiratory syndrome coronavirus-2 (SARS-CoV-2) invades host cells by interacting with multiple receptors and coreceptors [1,2,3,4,5]. The entry of SARS-CoV-2 into host cells is a dynamic process principally mediated by the viral spike (S) glycoprotein, which engages host cell receptors and co-receptors to trigger the fusion between the virus and cellular membrane fusion [6,7,8,9,10,11]. In a two-step proposed model for SARS-CoV-2 entry, the virus S glycoprotein first interacts non-specifically with the heparan sulfate (HS) for cell attachment [6,7,8]. In the second step, a more specific interaction between the primed S and the prototypic ACE-2 receptor results in virus–cell fusion [6,11]. Multiple other cell receptors and co-receptors such as neuropilin, CD147, DC-SIGN, c-lectin type receptors, Toll-like receptors, glucose-regulated protein 78, and cellular integrins have also been shown and/or proposed to be an alternative receptor route for virus entry [8,9,10,11]. However, as the S glycoprotein plays a key role in mediating viral entry, it is also the main antigenic determinant and the key target of neutralizing antibodies [11,12]. The efficacy of these neutralizing antibodies against S glycoprotein in decreasing disease incidence suggests that antivirals that target the early events in SARS-CoV-2 viral attachment and fusion would be a successful strategy to eliminate and/or reduce the disease [13,14].

A previous study utilizing SARS-CoV-2 pseudoviruses transduction in human embryonic kidney (HEK293T) cells demonstrated that entry was dependent on the ACE2 receptor and was sensitive to the pH of endosome/lysosome. Interestingly, the same study also found that the infection of SARS-CoV-2 pseudoviruses was cholesterol-rich and lipid raft-dependent. Therefore, we hypothesized that the presence of a cholesterol transporter, such as Niemann-pick type c1-like1 (NPC1L1), would increase SARS-CoV-2 cell entry. This hypothesis was supported by previous studies, which have demonstrated that numerous clinically relevant viruses use NPC1L1 during cell entry. Our experimental results clearly demonstrate that the co-expression of NPC1L1 together with the ACE2 receptor but not NPC1L1 alone synergistically increased the SARS-CoV-2 entry and cell-to-cell fusion, highlighting the significance of NPC1L1 in SARS-CoV-2 cell entry.

## 2. Results

**Figure 1:**
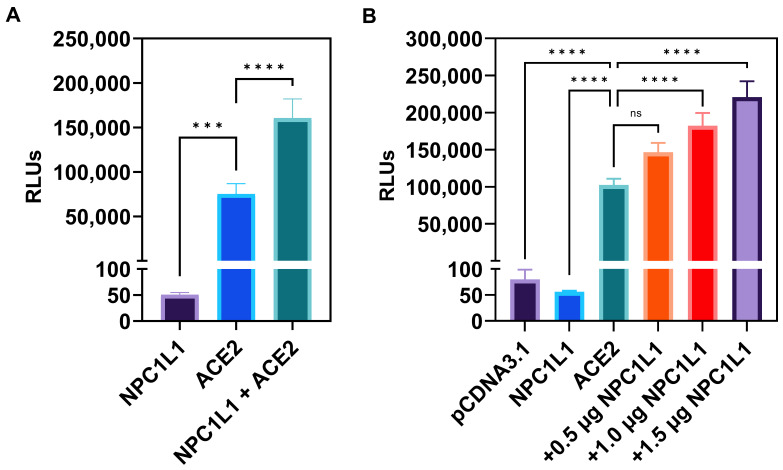
Chinese hamster ovary (CHO-K1) cells co-expressing human encoded NPC1L1 together with ACE2 significantly enhance SARS-CoV-2 cell entry. (**A**) CHO-K1 cells transfected either with NPC1L1 and pCDNA3.1, ACE2 and pCDNA3.1, or a combination of NPC1L1 and ACE2 were challenged with luciferase-expressing SARS-CoV-2 pseudovirus at 2 × 10^8^ RLU/mL. (**B**) CHO-K1 cells expressing NPC1L1 or ACE2 alone or co-expressing ACE2 with NPC1L1 in a concentration-dependent manner were challenged with luciferase-expressing SARS-CoV-2 pseudovirus at 2 × 10^8^ RLU/mL. CHO-K1 cells transfected with pCDNA3.1 were used as a negative control. RLU data in this experiment are expressed without TMPRSS2. Asterisks indicate a significant difference between the groups and ACE2 positive control (ns: not significant, *** *p* = 0.0005, **** *p* < 0.0001).

**Target cells co-expressing human ACE2 receptors with NPC1L1 but not NPC1L1 alone significantly enhances SARS-CoV-2 cell entry.** Since the entry pathway for SARS-CoV-2 uses a pH and cholesterol-dependent endosomal route [15], we first tested if the cholesterol uptake protein NPC1L1 impacts the entry of a pseudovirus expressing SARS-CoV-2 Spike (S) glycoprotein. Therefore, we used Chinese hamster ovary (CHO-K1) cells which do not express endogenous receptors and hence are resistant to SARS-CoV-2 cell entry. The CHO-K1 cells were independently transfected with human ACE-2 plasmid and the control empty vector (pCDNA3.1) plasmid. In parallel, CHO-K1 cells were transfected with NPC1L1 plasmid. Twenty-four hours post-transfection, the cells were transduced with a pseudovirus expressing the SARS-CoV-2 S glycoprotein (2 × 10^8^ RLU/mL). Forty-eight hours post-transduction, viral entry was determined by quantifying relative luciferase (RLU) activity. As shown in Figure 1A, NPC1L1 alone did not affect SARS-CoV-2 cell entry, while the target CHO-K1 cells expressing human ACE2 receptor clearly showed viral entry as evident from the positive luciferase signal. In addition, the co-expression of NPC1L1 with the human ACE2 receptor clearly boosted virus entry in comparison to cells only expressing the human ACE2 receptor. We next tested the dosage-dependent effects of NPC1L1 with human ACE2 receptors. In Figure 1B, CHO-K1 cells were co-transfected with 1 µg of human ACE2 plasmid DNA with increasing concentrations of NPC1L1 (0.5–1.5 µg). The data generated from this experiment clearly demonstrated a concentration-dependent enhancement in SARS-CoV-2 cell entry when the amount of NPC1L1 was increased.

**Figure 2:**
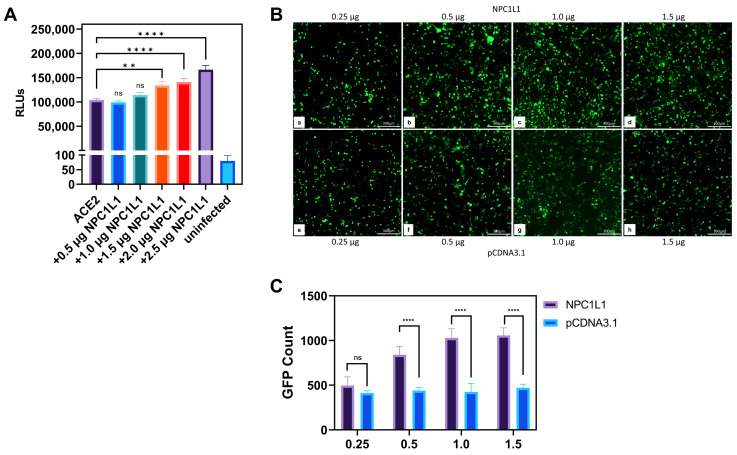
Human embryonic kidney (HEK293T-ACE2) cells co-expressing NPC1L1 together with human ACE2 further promote SARS-CoV-2 cell entry. (**A**) HEK293T-ACE2 cells expressing pCDNA3.1 or co-expressing both ACE2 and NPC1L1 in a concentration-dependent manner were challenged with reporter SARS-CoV-2 at 2 × 10^8^ RLU/mL. (**B**) HEK293T-ACE2 cells were transiently transfected with either NPC1L1 (panels a–d) and or pCDNA3.1 (panels e–h) in a dosage-dependent manner. The cells were then challenged with GFP expressing SARS-CoV-2 pseudovirus at 2 × 10^8^ VG/mL. GFP-positive cells were visualized as an indicator of viral entry. Microscopy was performed under 10× magnification using the ImageXpress Pico automated cell imaging system (Molecular Devices, San Jose, CA, USA). Scale bar 300 µm. (**C**) The estimated count of GFP-positive punctate was enumerated using ImageJ. First, the range of pixel density of GFP-positive punctate to be counted was established, and then the particle function analyzed was used to assign a numerical value. Once the range and image setting were established, the same parameters were used to analyze each subsequent image. Asterisks indicate a significant difference between the groups and ACE2 positive control (ns: not significant, ** *p* = 0.0018, and **** *p* < 0.0001).

**Human embryonic kidney (HEK293T) cells co-expressing NPC1L1 and human ACE2 receptors significantly promote SARS-CoV-2 entry.** Since CHO-K1 cells do not represent a true susceptible target cell line for SARS-CoV-2 entry, we next tested if the results generated in the CHO-K1 cells can be replicated with a human target cell line that stably expresses ACE-2. In this experiment, HEK293T-ACE2 cells were transiently transfected with pCDNA3.1 to serve as a positive control, while mock-transduced cells served as a negative control. Separately, HEK293T-ACE2 cells were transfected with various concentrations of plasmid encoding NPC1L1 (0.5–2.5 µg). The empty vector pCDNA3.1 was used to balance the concentration of DNA. After 24 h, cells were transduced with a reporter SARS-CoV-2 S glycoprotein-expressing pseudovirus and the luciferase activity was analyzed as described above. As shown in Figure 2A, a concentration-dependent enhancement in SARS-CoV-2 S glycoprotein-dependent cell entry was detected as the level of NPC1L1 plasmid DNA increased. This result clearly mimicked the above results generated using CHO-K1 cells, implicating the significance of NPC1L1 in SARS-CoV-2 pseudovirus entry in human cell lines.

**Figure 3:**
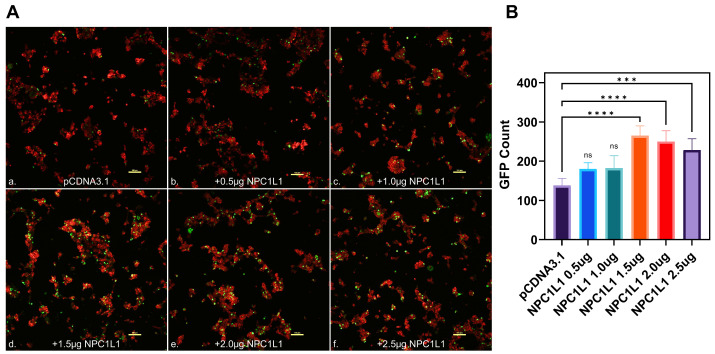
Visualization of SARS-CoV-2 entry into human embryonic kidney cells (HEK293T-ACE2) in the presence of NPC1L1. (**A**). Confocal imaging of HEK293T-ACE2 cells transfected with different concentrations of NPC1L1 (panels b–f) or pCDNA3.1 (panel a) before transduction with GFP expressing pseudotyped virus. The actin cytoskeleton was stained red with phalloidin, and pseudotyped SARS-CoV-2 infected cells showed a green GFP signal. Slides were imaged at 10× magnification using the Nikon A1R confocal microscope (Nikon Instruments, Melville, NY, USA), and images were processed using ImageJ. Scale bar 100 µm. (**B**). The estimated count of GFP-positive punctate was enumerated using ImageJ as previously described. Asterisks indicate a significant difference between the experimental and control groups (ns: not significant, *** *p* = 0.0004, and **** *p* < 0.0001).

**Imaging studies showed the dosage-dependent effect of NPC1L1 in promoting SARS-CoV-2 cell entry.** To visually confirm the NPC1L1/human ACE2 mediated enhancement for SARS-CoV-2 S cell entry, HEK293T-ACE2 cells were transfected with NPC1L1 in a concentration-dependent manner (0.25–1.5 µg). In parallel, the control HEK293T-ACE2 cells were also transfected in a similar dosage-dependent manner using pCDNA3.1. Twenty-four hours post-transfection, the cells were challenged with a GFP reporter expressing SARS-CoV-2 S glycoprotein pseudovirus at 2 × 10^8^ viral genes per mL for 2 h at 37 °C. Forty-eight hours post-infection, the cells were imaged for a green punctuate signal using fluorescent microscopy. As shown in Figure 2B (panels a–d), a concentration-dependent effect of NPC1L1 was visually evident as the amount of virus-associated GFP signal increased with the increasing concentration of NPC1L1. In contrast, a dosage-dependent effect for pCDNA3.1 did not enhance the SARS-CoV-2 associated GFP signal (Figure 2B; panels e–h). The green punctuate signals generated in this experiment were further quantified using ImageJ software (ver. 1.52a, NIH, Bethesda, MD, USA) that confirmed the positive impact of NPC1L1 in SARS-CoV-2 pseudovirus cell entry (Figure 2C). The above results were further verified using confocal imaging of the target cells expressing different concentrations of NPC1L1 transduced with GFP reporter expressing SARS-CoV-2 pseudovirus. As shown in Figure 3A, a dosage-dependent effect of NPC1L1 (panels b–f) on viral entry is clearly visible in HEK293T-ACE2 compared to the control HEK293T-ACE2 expressing pCDNA3.1 (panel a). The green punctuates were again quantified using ImageJ (Figure 3B), and the dosage-dependent effect appears to peak around 1.5 µg.

**Figure 4:**
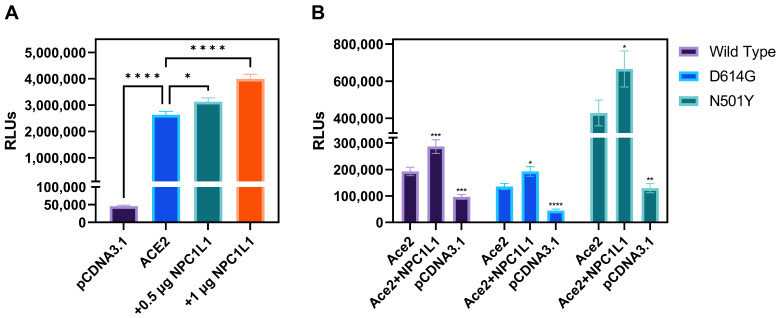
Target CHO-K1 cells co-expressing human encoded ACE2 and NPC1L1 significantly enhances SARS-CoV-2 spike glycoprotein-mediated cell fusion. (**A**) Effector CHO-K1 cells expressing S glycoprotein were co-cultured with the target cells co-expressing ACE2 and NPC1L1 or pCDNA3.1 with the luciferase gene. Membrane fusion as a means of virus cell-to-cell spread was detected by monitoring relative luciferase units (RLUs). (**B**) Cell-to-cell fusion using effector CHO-K1 cells expressing S glycoprotein from three different mutants; wild type: Wuhan-Hu-1, D614G mutant, and N501Y mutant. RLU data in this experiment are expressed without TMPRSS2. Asterisks indicate a significant difference between the groups and ACE2 positive control (* *p* = 0.0246, ** *p* = 0.0044, *** *p* = 0.0006, and **** *p* < 0.0001).

**Figure 5:**
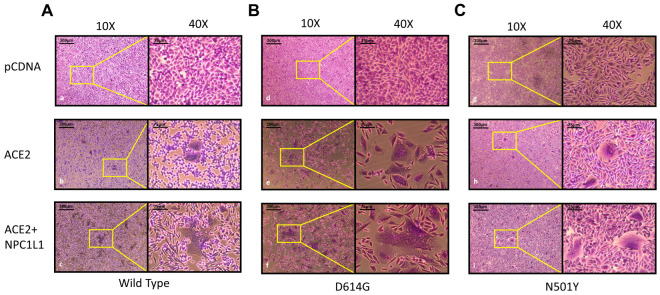
Impact of NPC1L1 expression on syncytia formation between effector and target cells using 3 different variant S glycoproteins; wild type: Wuhan-Hu-1 (panels a–c) (**A**), D614G mutant (panels d–f) (**B**), and N501Y mutant (panels g–i) (**C**). Images were captured under 10× and 40× magnification on EVOS FL auto imaging system after 24 h of co-culturing effector and target cells to highlight differences in size and abundance of syncytia in the presence and absence of NPC1L1.

**Effector cells expressing different variants of SARS-CoV-2 spike glycoprotein mediate strong cell-to-cell fusion with the target cell co-expressing NPC1L1 with the human ACE2 receptor.** We tested the impact of NPC1L1 using cell-to-cell fusion assays in which mechanistic interaction between the S glycoprotein and the host cell receptor (human ACE-2) leads to quantitative cell fusion. In this assay, the CHO-K1 cells co-expressing SARS-CoV-2 spike (S) glycoprotein with T7 RNA polymerase designated as an effector cell were co-cultured with the target CHO-K1 cells co-expressing human ACE2 and luciferase under the T7 promoter. As shown in Figure 4A, a significant increase in cell-to-cell fusion was observed when NPC1L1 was co-expressed in the target cells with the human ACE2 receptor. This effect was concentration-dependent since an increased concentration from 0.5 µg to 1.0 µg of NPC1L1 further enhanced S glycoprotein-mediated cell-to-cell fusion. In addition, we wanted to see if mutated versions of S glycoprotein were also impacted by NPC1L1 expression. As shown in Figure 4B, three different variations of S glycoprotein, i.e., wild-type Wuhan-Hu-1, D614G mutant, and N501Y mutant, showed greater cell-to-cell fusion when NPC1L1 was co-expressed along with ACE2 receptor. As expected, the relative luciferase units (RLUs) of pCDNA3.1 cells co-cultured with the effector cell expressing spike glycoprotein were significantly lower than the positive ACE2 target cells co-cultured with the effector cells expressing spike glycoprotein because target pCDNA3.1 cells lacked ACE-2 receptor, which is the key receptor used by the SARS-CoV-2 during cell entry and cell fusion [6]. This trend was also verified to determine if NPC1L1 expression would impact SARS-CoV-2 spike glycoprotein-mediated syncytia formation. The effector cells expressing S spike glycoprotein were co-cultured with target cells expressing ACE2 and or ACE2 with NPC1L1 for 24 h post mixing. As shown in Figure 5, much larger and more abundant syncytia formation were visible in all variants tested when NPC1L1 is expressed compared to ACE2 alone. We also confirmed the expression of NPC1L1 in CHO-K1 cells prior to pseudovirus and cell-to-cell fusion experiments (Supplementary Figure S1).

**Overexpression of NPC1L1 strongly enhances live SARS-CoV-2 entry into the target cells.**

**Figure 6:**
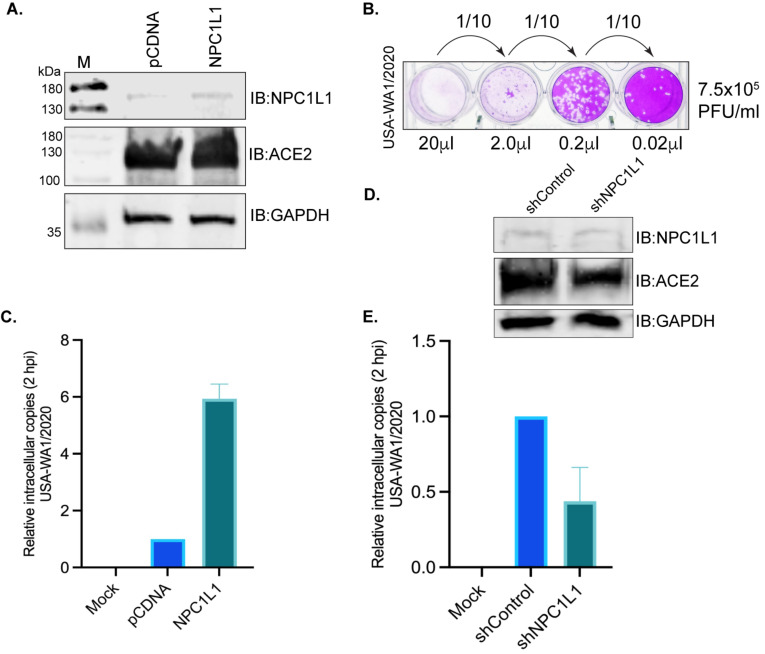
NPC1L1 enhanced the entry of the SARS-CoV-2 virus in HEK 293T-ACE2 cells. (**A**) HEK 293T-ACE2 cells transfected with 2 μg of pCDNA or NPC1L1 expression plasmid using Lipofectamine 2000 were harvested 48 h post-transfection for the detection of NPC1L1 expression using anti-NPC1L1 antibody. The membrane was also immunoblotted with an anti-ACE2 antibody for the detection of ACE2 expression. GAPDH immunoblot was used as a housekeeping loading control. M- molecular weight marker; pCDNA-lysates from cells transfected with empty vector, pCDNA; NPC1L1-lysates from cells expressing NPC1L1. (**B**) Plaque assay for estimating the live SARS-CoV-2 (USA-WA1/2020) virus through plaque forming units (PFU) in the stock used for infection assays. A total of 20 μL of the original stock and 10-fold subsequent dilution was added to the monolayer of VERO E6-TMPRSS2-T2A-ACE2 for estimating the number of PFU per mL. (**C**) A total of 50 μL of the USA-WA1/2020 original stock (~3.75 × 10^4^ PFU) was used for infecting a monolayer of HEK 293T-ACE2 cells (~200,000 cells/well of a 12-well plate) transfected with pCDNA or NPC1L1 expression plasmid for 48 h. Un-transfected and un-infected cells were used as mock control. Total RNA extracted following 2 h incubation with the virus at 37 °C, after removing any unattached virus, was used for the quantitation of internalized virus following infection. RNA from three independent infection assays were used for the quantitation of the viral genome using SARS-CoV-2 RUO qPCR Primer & Probe Kit on Quant studio 5 with TaqPath™ 1-Step RT-qPCR Master Mix. Human GAPD (GAPDH) endogenous control (FAM™/MGB probe) was used for normalization and relative quantitation of SARS-CoV-2 in cells expressing NPC1L1 compared to control, pCDNA cells. HEK 293T-ACE2 cells expressing NPC1L1 showed an enhanced infection with SARS-CoV-2. Mock cells did not show any detectable levels of the SARS-CoV-2 genome, as expected. (**D**) HEK 293T-ACE2 cells were subjected to NPC1L1 depletion using lentiviral particles and control shRNA lentiviral particles. The depletion of NPC1L1 was confirmed by immunoblot with anti-NPC1L1 antibody along with the detection of ACE2 and GAPDH with respective antibodies. (**E**) NPC1L1-depleted and control cells were infected with SARS-CoV-2 USA-WA1/2020 original stock (~3.75 × 10^4^ PFU) using a monolayer setup. Un-infected cells served as the mock control. Total RNA was extracted following a 2 h incubation with the virus at 37 °C, subsequent to the removal of any unattached virus. The quantitation of internalized virus post-infection was performed using RNA from the independently infected replicates. The SARS-CoV-2 RUO qPCR Primer & Probe Kit on Quant Studio 5 with TaqPath™ 1-Step RT-qPCR Master Mix was employed for the quantitation of the viral genome. Human GAPD (GAPDH) endogenous control (FAM™/MGB probe) was utilized for normalization and relative quantitation of SARS-CoV-2 in NPC1L1-depleted cells compared to control cells (sh-Control). HEK 293T-ACE2 cells with NPC1L1 depletion exhibited a marked reduction in SARS-CoV-2 infection compared to control cells.

We tested whether the NPC1L1 expression would impact SARS-CoV-2 cell entry into the target cells. We used the USA-WA1/2020 variant of SARS-CoV-2 for infection assays in a BSL-3 laboratory. HEK293T-ACE2 cells that were transfected with NPC1L1 expression plasmid showed a detectable level of NPC1L1 compared to the empty vector-transfected cells (Figure 6A). The number of infectious SARS-CoV-2 (USA-WA1/2020) in our stock was estimated by standard plaque assay on VERO E6-TMPRSS2-T2A-ACE2 cells by using 20 μL of the original stock as well as 10-fold dilutions to quantify the plaque forming units (PFU) accurately. Based on the number of plaques in replicates, we estimated ~7.5 × 10^5^ PFU/mL in our stock. A representative plate of dilution series and plaques is presented in Figure 6B. We infected the monolayer of HEK293T-ACE2 cells expressing NPC1L1 or vector with a 50 μL (~3.75 × 10^4^ PFU) of the above virus stock and estimated the number of intracellular virus after 2hpi after removing any loosely bound virus on the cell surface. An estimation of the intracellular SARS-CoV-2 genome among these groups showed successful infection of these cells when compared with the mock-infected cells (Figure 6C). Furthermore, the relative estimation of the SARS-CoV-2 genome in cells expressing NPC1L1 showed a significantly higher (~6-fold) number of viral genomes compared to the cells transfected with the vector, pCDNA (Figure 6D). This confirmed that the expression of NPC1L1 can further enhance the entry of SARS-CoV-2 in ACE2-expressing cells.

**Depletion of NPC1L1 diminishes the entry of SARS-CoV-2 into the target cells.**

Finally, we performed a SARS-CoV-2 infection assay in NPC1L1-depleted 293T-ACE2 cells to determine the impact of NPC1L1 on virus entry. Analysis of the intracellular virus copies in NPC1L1-depleted 293T-ACE2 cells showed a significant reduction in comparison to the control cells without NPC1L1 depletion (Figure 6E). These findings strongly suggest that the depletion of NPC1L1 has a pronounced effect on diminishing the cellular entry of SARS-CoV-2 in HEK293T-ACE2 cells, emphasizing the crucial role of NPC1L1 in facilitating the virus’s entry into host cells.

## 3. Discussion

SARS-CoV-2 exploits cholesterol-rich lipid rafts as part of the viral entry process [16], and NPC1L1 is an integral component in lipid rafts and acts as a vesicular endosomal receptor; therefore, we first tested if NPC1L1 enhances SARS-CoV-2 cell entry. In fact, previous studies have shown that pseudovirus-based SARS-CoV-2 transduction in HEK293T cells depends on pH- and cholesterol-rich lipid rafts [17,18]. Similarly, a different group had also demonstrated the significance of lipid rafts in SARS-CoV entry using VeroE6 cells [19]. Our data demonstrated that the presence of NPC1L1 significantly enhances ACE-2-mediated SARS-CoV-2 cell entry and S glycoprotein-mediated cell-to-cell fusion. In contrast, NPC1L1 alone had no effect on viral entry and cell-to-cell fusion. Clearly, our finding provides the significance of NPC1L1 as an important vesicular protein which could potentially also be aiding in virus entry and transport to promote cell infectivity. However, future studies are needed to understand the NPC1L1-driven mechanism including cholesterol uptake and aiding in SARS-CoV-2 cell entry. Since cholesterol is a major lipid component of lipid rafts, the influence of NPC1L1 in maintaining the integrity and functions of cell membranes, including receptor-mediated endocytosis, virus trafficking, and the associated signaling, needs further investigation.

The scientific relevance to test NPC1L1 in SARS-CoV-2 entry was also because multiple other viruses such as Ebola, human immunodeficiency virus, Hepatitis C virus, Dengue, Chikungunya, and Zika virus use NPC1L1 either during cell entry and/or during late stages of virus trafficking [20,21,22,23,24,25,26,27,28]. In the case of the Hepatitis C virus, the NPC1L1 receptor serves as a potential tropism determinant since it is highly expressed in hepatocytes [23,24]. Interestingly Ebola virus binding to NPC1L1 triggers the priming of the viral glycoprotein into a fusion-competent state resulting in virus cell membrane fusion [22,23]. In fact, the physiological relevance of NPC1L1receptor has been directly connected to an enhanced cholesterol uptake during Dengue virus infection, and the drug ezetimibe inhibits Dengue virus infection in the human hepatoma cell line (Huh-7) by blocking NPC1L1 [27,28]. Finally, compounds interfering with the intracellular cholesterol transport, which also inactivates late endosome and lysosome formation, inhibit Chikungunya and Zika virus infection [25,26]. However, in our results, NPC1L1 had no activity alone for cell entry, but the co-expression of NPC1L1 with human ACE-2 receptor dramatically increased the viral entry. Our data demonstrated that the overexpression of NPC1L1 increases SARS-CoV-2 entry and has significant clinical relevance. Previous studies indicate that obese individuals not only have increased ACE2 receptor levels [29] but also higher levels of NPC1L1 [30]. It is interesting to speculate, based on our data, that obese individuals are at increased risk for severe COVID-19 disease in part because of the additive effects of higher levels of ACE2 receptor and NPC1L1 in adipose tissue. In support of this idea, obese individuals have been shown to have a higher viral load [29], suggesting that these factors have a major impact on disease course as well. Interestingly, obese individuals also have an altered immune response to SARS-CoV-2 [31]. This could be due in part to the contributions of adipocytes during the SARS-CoV-2 response. If adipocytes are directly infected by SARS-CoV-2, as has been suggested [32], this could directly impact the production of pro-inflammatory cytokines through adipocytes [33] or indirectly promote inflammation by altering the adipocyte metabolism, thereby stimulating inflammation by cells of the immune system [33]. Therefore, the enhanced expression of ACE2 and NPC1L1 on adipose tissues could result in enhanced adipocyte infection that contributes to increased inflammation and mortality in SARS-CoV-2 infections.

Although the role of NPC1L1 during SARS-CoV-2 infection is currently emerging [34,35], the precise role of NPC1L1 remains poorly defined during cell entry. It has been predicted that SARS-CoV-2 infection in NPC1L1 deficient cells influences the stability of ACE2 and TMPRSS2, which reside within these membranes [36]. Interestingly, NPC1L1 is endogenously expressed in the apical membrane of the gastrointestinal tract, the canalicular membrane of the liver, appendix, and kidneys but not in the epithelial cells of the airways [35,36,37,38,39]. In fact, a clinical study presented direct evidence of SARS-CoV-2-associated gastrointestinal infection in a COVID-19 patient, which caused acute hemorrhagic colitis [40]. Interestingly, NPC1L1 knockout mice showed protective effects against colitis-associated tumorigenesis by decreasing plasma lipids, especially cholesterol, to reduce inflammation and β-catenin, p-c-Jun, and p-ERK signaling [41]. Taken together, the observed role of NPC1L1 in promoting SARS-CoV-2 entry into human kidney cells is not only highly intriguing but also quite relevant. This relevance stems from the fact that NPC1L1 and ACE2 exhibit high expression levels in the kidneys [35]. Previous studies have also confirmed that kidneys are directly impacted by SARS-CoV-2, thereby resulting in acute kidney injury in COVID-19 patients and the development of chronic kidney disease in long COVID [42,43,44,45]. While our data provide insights into SARS-CoV-2 entry in the human embryonic kidney cell model, the contribution of NPC1L1 and other factors potentially resulting in acute cell injury and associated inflammatory damage require further investigation. The usage of drugs, such as ubiquinone, ezetimibe, and rosuvastatin, that target cholesterol synthesis pathways has already shown great promise in reducing the severity of COVID-19 and the hospitalization risk [46]. Therefore, future mechanistic understanding regarding NPC1L1-mediated cell signaling pathways during SARS-CoV-2 entry would certainly generate novel targets to prevent new infection.

## 4. Materials and Methods

### 4.1. Cell Culture

Chinese hamster ovary cells (CHO-K1, ATCC, Manassas, VA, USA), human kidney epithelial cells expressing ACE2 (HEK293T-ACE2, Genecopoeia, Rockville, MD, USA), human lung adenocarcinoma cells (Calu-3, ATCC), and African green monkey kidney cells expressing TMPRSS2 and ACE2 (Vero E6-TMPRSS2-T2A-ACE2, BEI Resources, NIAID/NIH, Bethesda, MD, USA) were used for in vitro studies. Cells were grown at 37 °C with 5% CO₂ and were passaged according to the manufacturer’s recommendations using 0.5% trypsin EDTA after reaching near-confluence. CHO-K1 cells were grown in Ham’s F-12 medium supplemented with 10% fetal bovine serum (FBS) and 100 U/mg/mL penicillin/streptomycin (P/S). HEK293T-ACE2 cells were grown in Dulbecco’s modified Eagle medium (DMEM) supplemented with 100 U/mg/mL P/S, 100 µg/mL hygromycin B, and 10% FBS. Vero E6-TMPRSS2-T2A-ACE2 were cultured in DMEM supplemented with 10% FBS, 2 mM L-glutamine, 100 U/mg/mL P/S, and 10 µg/mL puromycin. Calu-3 cells were grown in DMEM supplemented with 100 U/mg/mL P/S and 10% FBS.

### 4.2. Pseudoviruses, SARS-CoV-2 Virus, and Expression Plasmids

D614G spike-pseudotyped lentivirus (Cat # SP003-100) was purchased from Genecopoeia and was used for luciferase-based studies. Baculovirus vector pseudotyped with spike M2 green reporter (Cat # C1122G) was purchased from Montana Molecular and was used for imaging studies. SARS-Related Coronavirus 2, Isolate USA-WA1/2020, was deposited by the Centers for Disease Control and Prevention and obtained through BEI Resources, NIAID, NIH: SARS-Related Coronavirus 2, Isolate USA-WA1/2020, NR-52281. P1 virus stock was prepared from the ATCC/BEI stock by inoculating a monolayer of Calu-3. Four days later, the Calu-3 monolayer showed a typical cytopathic effect due to SARS-CoV-2 infection with supernatant-containing virions collected for infection assays following centrifugation to remove any cell debris. The number of infectious viruses and plaque-forming units (PFU) was estimated by plaque assay following a serial dilution of collected supernatant. The mammalian expression plasmids encoding SARS-CoV-2 spike glycoprotein D614G (Cat # VG40589-UT), N501Y (Cat # VG40771-UT), and human ACE2 receptor (Cat # HG10108-UT) were purchased from Sino Biological (Wayne, PA, USA). The vector containing SARS-CoV-2 Wuhan-Hu-1 spike glycoprotein (Cat # NR-52310) was purchased from BEI Resources, NIAID, NIH. The vector containing NPC1L1 was a gift from RESOLUTE Consortium & Giulio Superti-Furga (Addgene Cat # 132303). The luciferase reporter plasmids (pCAGT7 Pol and pT7EMCLuc) used in the cell-to-cell fusion assay were a gift from Professor Shukla’s lab (University of Illinois at Chicago, IL, USA).

### 4.3. SARS-CoV-2 Spike Protein-Pseudoypted Lentivirus Entry Assay

HEK293T-ACE2 or CHO-K1 cells were grown to 70–80% confluence in a 6-well plate in DMEM or F12 supplemented with 10% heat-inactivated FBS. The following day, CHO-K1 cells were co-transfected with 1 µg ACE2 and 0.5–1.5 µg NPC1L1, and 293-T cells were transfected with 0.5–2.5 µg of NPC1L1. As a control, separate wells were transfected with NPC1L1, ACE2, and empty vector pCDNA3.1. After 24 h the transfection media was removed, and cells were transferred to a 96-well plate. Following overnight growth, media was replaced with 100 µL of fresh media containing 0.5 µL pseudovirus per well, and the plate was incubated for 2 h at 4 °C with gentle rocking to increase transduction efficiency. The plate was then moved to 37 °C for 48 h. After 48 h, the media was removed and 30 µL/well of reporter lysis buffer was added. The plate was rocked for 30 min, treated to a freeze–thaw at −80 °C, and 20 µL of lysate was transferred to a white 96-well luciferase assay plate. A total of 50 µL of luciferase assay reagent was added, the plate was mixed, and readout was collected using the EnSpire Multimode Plate Reader (PerkinElmer) at a speed of 0.5 s per well.

### 4.4. Fluorescent Microscopy

HEK293T-ACE2 cells were grown on a 12-well plate and transfected with 0.25–1.5 µg of NPC1L1 or empty vector pCDNA 3.1 as previously described. Following transfection, the media was replaced with 500 µL DMEM media containing 20 µL of pseudotyped virus and 2 µL of 500 mM sodium butyrate, as per the manufacturer’s recommendations. The plate was incubated at 4 °C with gentle rocking for 2 h to increase viral binding before being transferred to 37 °C for 24 h. After 24 h, the wells were gently washed with PBS, and the cells were fixed in 4% paraformaldehyde for 20 min. Fixed cells were washed 3× with PBS before imaging on the ImageXpress Pico automated cell imaging system (Molecular Devices, San Jose, CA, USA). Images were then processed and GFP-positive cells were enumerated using ImageJ (version 1.52a, NIH, Bethesda, MD, USA).

### 4.5. Confocal Imaging

HEK293T-ACE2 was grown on poly-L-lysine coated cover glass overnight in a 6-well plate. The following day, cells were transiently transfected with NPC1L1 (0.5 µg–2.5 µg), balanced to 2.5 µg DNA per well with an empty vector DNA (pCDNA3.1), and the controls received 2.5 µg of pCDNA3.1 alone. Twenty-four hours post-transfection, the media was replaced with 1 mL DMEM media containing 3.3 × 10^7^ VG/mL of pseudotyped virus and 2 mM of sodium butyrate, as per the manufacturer’s recommendation. The plate was gently rocked for 2 h at room temperature before being transferred to 37 °C for 24 h. After 24 h, the cells were gently washed with PBS and were fixed in 4% paraformaldehyde for 20 min. Following fixation, the cover glass was washed 3× in PBS and then incubated with phalloidin (Thermo Fisher, Waltham, MA, USA) at a ratio of 1:40 in a blocking buffer for 30 min. The cover glass was washed an additional 3× in PBS with a final rinse in sterile water before mounting to slides using HardSet mounting media without DAPI (Vector Laboratories, Newark, CA, USA). Slides were imaged at 10× magnification using the Nikon A1R confocal microscope (Nikon Instruments, Melville, NY, USA) and images were processed using ImageJ (version 1.52a, NIH, Bethesda, MD, USA).

### 4.6. Split Luciferase Cell-to-Cell Fusion Assay

Virus glycoprotein cells were considered as the “effector cells” while the cell expressing entry ACE2 receptor was considered as the “target cell”. The terminology effector and target cells have been frequently used in various cell-to-cell fusion assays, including in the SARS-CoV-2 field [47,48,49]. Effector CHO-K1 cells expressing virus glycoprotein were seeded on 6-well plates at a density not exceeding 90% confluency after overnight growth in Hams F12 media supplemented with 100 U/mg/mL P/S and 10% FBS. The following day, effector cells were co-transfected with 1 µg SARS-CoV-2 spike protein (Wuhan-Hu-1, D614G, or N501Y) and 500 ng of T7 RNA polymerase per well. Target cells were co-transfected with 1 µg of ACE2 receptor, 500 ng of luciferase, and 500 ng or 1 µg of NPC1L1 per well in Opti-MEM serum-free media (Gibco) using lipofectamine 2000 (Invitrogen). To account for background and baseline fusion activity, control target wells were co-transfected with empty vector pCDNA 3.1 and luciferase. After 24 h, the transfection mix was removed, and cells were washed with PBS. Effector and target cells were lifted using 500 µL trypsin, resuspended in 1.5 mL complete media, and equal volumes of effector and target cells were transferred to a 12-well plate and incubated for 24 h to allow fusion to occur. After 24 h, the media was removed, wells were washed with PBS and were either lysed with 120 µL/well of reporter lysis buffer (Promega, Madison, WI, USA) or fixed in 4% PFA and stained with Giemsa (Sigma-Aldrich, St. Louis, MO, USA) for microscopy using the EVOS FL auto imaging system (ThermoFisher). Plates were rocked for 30 min, and cells were scraped and freeze-thawed to achieve complete cell lysis. In total, 20 µL of cell lysate was then transferred to clear culture tubes, 50 µL luciferase assay reagent (Promega) was added, and luciferase signal was recorded using FB12 single tube luminometer (Berthold, Bad Wildbad, Germany).

### 4.7. Transfection of 293T-ACE2 Cells and Immunoblotting

293T-ACE2 cells were seeded at a density of 200,000 cells per well in 12-well plates and incubated overnight. For each transfection, 2 μg of pCDNA or NPC1L1 plasmids were used. Quadruplicate transfections were performed for each condition. After 48 h of transfection, cells were trypsinized, harvested, and collected. The collected cells were then re-plated at a density of 200,000 cells per well in 12-well plates for SARS-CoV-2 infection assays by transferring these plates to the BSL-3 laboratory. A fraction of cells (500,000) transfected with the above plasmids were subjected to a Western blot analysis for the detection of NPC1L1 and ACE2 levels. For the Western blot assay, cells were washed with cold PBS (1×) and lyses in radioimmunoprecipitation assay (RIPA) lysis buffer (1% NP-40, 50  mM Tris [pH 7.5], 1  mM EDTA [pH 8.0], and 150  mM NaCl) complemented with protease inhibitors (1  mM phenylmethylsulfonyl fluoride [PMSF], 1  μg/mL aprotinin, 1  μg/mL pepstatin, 1  μg/mL sodium fluoride, and 1  μg/mL leupeptin). After 30 min lysis on ice, cell debris was removed via centrifugation (12,000  rpm; 10 min at 4 °C). Clear lysate was mixed with the 3× loading dye and boiled at 95 °C for 5 min. Subsequently, proteins were resolved by SDS-polyacrylamide gel electrophoresis and transferred via Western blotting onto a 0.45-μm nitrocellulose membrane (Bio-Rad Laboratories, Hercules, CA, USA) using established protocols. To detect the proteins of interest, ACE2 (ThermoFisher cat. MA532307), NPC1L1 (ThermoFisher cat. PA116800), and GAPDH (ThermoFisher cat. PA1987) antibodies were applied to the membrane and incubated overnight at 4 °C. This was followed by incubation with appropriate infrared-dye-tagged secondary antibodies (IR-Dye800) and scanning using a Bio-Rad scanner. CHO-K1 cell lysates for the demonstration of NPC1L1 expression in pseudovirus experiments were prepared and blotted similarly and proteins of interest were detected using anti-NPC1L1 (Proteintech cat. 28085-1-AP) and anti-beta-Actin (Novus Biological cat. NBP1-47423).

### 4.8. Depletion of NPC1L1 in 293T-ACE2 Cells

NPC1L1 was depleted using lentiviral particles expressing shRNA for NPC1L1 (cat. # sc-61225-V) or control shRNA (cat. # sc-108080) from Santa Cruz Biotechnology, Inc. Cell monolayers of 293T-ACE2 were transduced with the lentiviral particles as per the manufacturer’s instruction (Santa Cruz Biotechnology, Dallas, TX, USA) and selected with puromycin (10 mg/mL) to obtain the cells expressing shRNA. Depletion of NPC1L1 was confirmed by collecting a fraction of sh-NPC1L1 and sh-Control cells and performing Western blotting for NPC1L1, ACE2, and GAPDH, as described above.

### 4.9. SARS-CoV-2 Infection Assays

293T-ACE2 cells expressing NPC1L1 or control vector (pcDNA) as well as NPC1L1 depleted (sh-NPC1L1) or control (sh-Control) cell monolayer were infected with 50 µL (~3.75 × 10^4^ PFU) of the USA-WA1/2020 stock per well of the 12-well plates in Biological Safety Level 3 (BSL-3) containment facility (University of Nevada, Reno, NV, USA). The monolayer of un-transfected 293T-ACE2 cells was used as a negative (mock) control. These infected cells were incubated at 37 °C in a CO_2_ incubator for 1 h for virus attachment and entry. Following incubation, the medium containing the virus was removed, and the cell monolayer was washed with PBS (1×), followed by mild trypsinization to remove any loosely bound SARS-CoV-2. Cells were washed once more with 1 ml of complete medium to remove residual trypsin and incubated with DMEM with 10% FBS and P/S for an additional 2 h to continue infection. Intracellular SARS-CoV-2 virus copies were determined by harvesting the cells following the removal of the medium and washing them with 1× PBS. Total RNA was extracted by lysing the cells in Trizol-LS (ThermoFisher Scientific) and using Direct-Zol RNA Miniprep Kits (Zymo Research, Irvine, CA, USA). Intracellular SARS-CoV-2 genome copies were determined using SARS-CoV-2 RUO qPCR Primer & Probe Kit (IDT, Coralville, IA, USA) on Quantstudio 5 with TaqPath™ 1-Step RT-qPCR Master Mix (ThermoFisher Scientific). Human GAPD (GAPDH) Endogenous Control (FAM™/MGB probe cat. #4352934E, ThermoFisher Scientific) was used as a normalizer for estimating a relative copy of the SARS-CoV-2 in the cells using the ∆∆ Ct method.

## 5. Statistical and Software Analysis

All the experiments were carried out at least in triplicate unless otherwise noted. GraphPad Prism 9 was used to analyze data for statistical significance (*p* < 0.05). One-way analysis of variance (ANOVA) was used to determine significance between control and experimental groups followed by Bonferroni’s multiple comparisons test to determine significance compared to the positive control. ImageJ was used to assemble images and count GFP positive punctate. In all figures, the columns represent the mean of the data collected and the error bars represent SD. (****) signifies a *p*-value of <0.0001.

## Data Availability

The data presented in this study are available on request from the corresponding author.

## Supplementary Materials

The following supporting information can be downloaded at: https://www.mdpi.com/article/10.3390/biomedicines12040821/s1, Figure S1: NPC1L1 expression in CHO-K1 cells. CHO-K1 cells transfected with NPC1L1 or pCDNA using Lipofectamine 2000 were harvested 24 h post-transfection to confirm the expression of NPC1L1 for pseudovirus and cell-to-cell fusion experiments. Β-Actin was used as a loading control.
