# Supplementary figures and images for: Co-Expression of Niemann-Pick Type C1-Like1 (NPC1L1) with ACE2 Receptor Synergistically Enhances SARS-CoV-2 Entry and Fusion
Source: Biomedicines. 2024 Apr 8;12(4):821. doi: 10.3390/biomedicines12040821 (PMC11048565; doi:10.3390/biomedicines12040821)

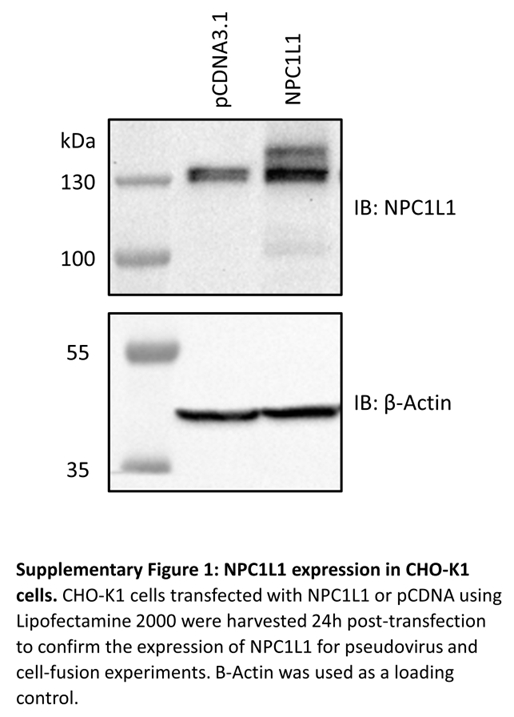

Supplement: Supplementary file 1 [file biomedicines-12-00821-s001.zip › biomedicines-2907705-supplementary.jpg]
